# Role of admission cardiotocography in predicting the obstetric outcome in term antenatal women: A prospective observational study

**DOI:** 10.34763/jmotherandchild.20222601.d-22-00017

**Published:** 2022-11-02

**Authors:** Naina Kumar, Monu Yadav

**Affiliations:** Department of Obstetrics and Gynecology, All India Institute of Medical Sciences, Bibinagar-508126, Hyderabad, Telangana, India; Department of Obstetrics and Gynecology, Maharishi Markandeshwar Institute of Medical Sciences and Research, 133207, Ambala, Haryana, India

**Keywords:** Antenatal, cardiotocography, fetal heart rate, fetal hypoxia, perinatal

## Abstract

**Background:**

Admission cardiotocography (CTG) used for fetal heart rate monitoring is an easy, cost-effective, non-invasive screening method for fetal well-being.

**Objectives:**

To evaluate the role of admission CTG in predicting fetal hypoxia in term antenatal women during labour ward admission and to correlate the results with perinatal and maternal outcomes.

**Material and methods:**

The present prospective observational study was conducted in the Obstetrics and Gynecology Department of the rural tertiary centre in Northern India over one year on 100 term antenatal women admitted to the labour ward in the first stage of labour. Participants were subjected to admission CTG for 20 minutes, and the pattern of fetal heart rate (reactive, suspicious, ominous) was recorded. Perinatal and maternal outcomes were assessed based on the fetal heart rate pattern on the admission CTG.

**Results:**

Of 100 term antenatal women, 51 were low-risk and 49 were high-risk cases. In the low-risk group, 92.16% had reactive traces, 3.92% suspicious and 3.92% ominous traces on admission CTG, whereas, in the high-risk group, 40.81% had reactive, 32.65% suspicious and 26.54% ominous tracing (p < 0.0001). The most common mode of delivery in both groups with ominous tracing was cesarean section (p = 0.0001). Abnormal CTG was significantly associated with adverse perinatal outcomes including poor one-minute Apgar scores (p < 0.05), need for resuscitation and NICU admission (p < 0.05). The specificity and negative predictive value of admission CTG in low- and high-risk groups were 97.9% and 93.6%, and 85.0% and 85.0%, respectively.

**Conclusion:**

Admission CTG is an effective, inexpensive, non-invasive technique to detect fetal hypoxia in low-and high-risk pregnancies in developing countries with an increased workload.

## Introduction

Motherhood is a dream of every woman, and the ultimate goal of having a healthy mother and a healthy baby is the responsibility of every obstetrician [1]. Fetal surveillance during labour is a crucial component for ensuring the delivery of a healthy baby with minimal intervention [2]. Furthermore, labour poses physiological stress to the fetus. During uterine contraction, there is a 60% reduction in uteroplacental perfusion, resulting in transient fetal and placental hypoxia. Most healthy fetuses tolerate this amount of hypoxia by peripheral chemoreflex activation, which in turn causes the redistribution of oxygenated blood to critical organs, including the heart, brain and adrenals [3], but not all fetuses are fortunate enough to tolerate this stress. Hence, continuous intrapartum electronic fetal heart rate monitoring is very essential to identify such fetuses at risk of developing fetal hypoxia and its related complications [4]. Cardiotocography (CTG) is one such way of assessing fetal well-being, as it records fetal heart rate (FHR), fetal movements and uterine contraction patterns to know the cause of hypoxia [5]. Admission CTG can be used for triaging fetuses at risk of hypoxia by recording FHR and uterine activity over 20 minutes during admission of term antenatal women in the labour ward [6], as early diagnosis can help in preventing many long-lasting fetal complications due to hypoxia through timely intervention. Hence, the present study was conducted with the aim to evaluate the role of admission CTG in predicting fetal hypoxia in term antenatal women and to correlate the results with the overall perinatal and maternal outcomes.

## Material and methods

**Study Design, Setting and Duration**: A prospective observational study involving term (≥ 37 completed weeks) antenatal women admitted to the labour ward of the Department of Obstetrics and Gynaecology of a rural tertiary care centre in Northern India over one year (January 2017–January 2018).

### Participants

**Inclusion criteria**: All pregnant women (primigravida/ multigravida) with live, singleton, term pregnancy (gestation ≥ 37 weeks) with cephalic presentation admitted to the labour ward in their first stage of labour (spontaneous in onset) were included as study participants. They were divided into high- and low-risk cases based on the presence or absence of risk factors, including anemia, hypertensive disorders of pregnancy, Rh isoimmunization, medical disorders (diabetes, thyroid disorders, renal disease), post-dated, oligo/polyhydramnios, fetal growth restriction and prelabour rupture of membranes.

**Exclusion criteria**:

– Pregnant women with gestation < 37 weeks;
– multifetal gestation;
– lie or presentation other than cephalic;
– dead or congenitally malformed fetus;
– history of previous cesarean section;
– with abruption;
– cord prolapse;
– or uterine rupture;
– those reporting in the second stage of labour; use of sedative in mothers 24 hours before testing and those refusing to participate were excluded from the study.

**Figure:**
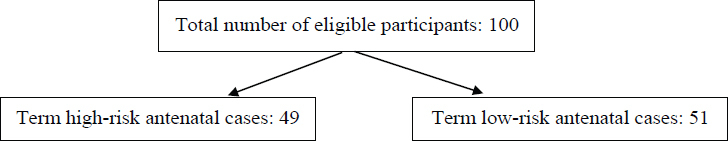


**Sampling Procedure**: Consecutive sampling of participants fulfilling the inclusion criteria was done for the entire duration of the study.

**Data collection**: After informed written consent from all the participants in their vernacular language, various sociodemographic parameters such as age, religion, occupation, registration status, gravidity, parity, number of live children and current gestational age were recorded on a preformed data collection sheet by trained nursing staff. A detailed medical, surgical and family history of all the participants was recorded, followed by a thorough physical examination including abdominal and vaginal examination to confirm the gestation, presentation and stage of labour. Depending on the presence or absence of high-risk factors mentioned in the inclusion criteria, the participants were divided into two groups: Group I – Term antenatal women with high-risk pregnancy and Group II – Term antenatal women with low-risk pregnancy. The participants in both groups were then subjected to admission CTG for 20 minutes at a speed of 1cm/min. It was done in a separate room with the patient lying in a semi-lateral position. The FHR tracings thus obtained were categorised according to the National Institute for Clinical Excellence clinical guidelines [7] as:

- Normal/Reactive/Reassuring: Baseline FHR 110–160 beats per min (bpm), or baseline variability > 5bpm or absence of any decelerations or at least two accelerations (>15 bpm for >15 sec) in 20 minutes duration.
- Suspicious/equivocal: Moderate tachycardia (161–180 bpm)/ bradycardia (100–109 bpm) or reduced baseline variability (< 5bpm) for > 40 mins but < 90 mins, although baseline heart rate remains normal (110–160 bpm) or presence of deceleration (early/variable/late).
- Ominous/Pathological: Baseline FHR >180 bpm or < 100 bpm or Sinusoidal pattern >10 mins (regular oscillation of baseline FHR with the absence of long-term variability) or baseline variability (< 5 bpm) for > 90mins.

Women with reactive tracing were monitored by either intermittent auscultation every 30 minutes in the first stage and every 5 minutes in the second stage of labour or with continuous cardiotocograph. The women with suspicious tracing were monitored with continuous cardiotocograph, and any abnormal FHR tracing with significant decelerations (variable or prolonged deceleration), baseline FHR < 110bpm or > 160bpm with loss of variability, were considered as having fetal distress and were delivered by either instrumental delivery or by emergency cesarean section. The colour of the liquor, mode of delivery, neonatal Apgar scores at one and five minutes, neonatal birth weight, need for neonatal resuscitation and neonatal intensive care unit (NICU) admission were recorded in the datasheet. Results of the admission CTG test were then compared with various labour outcome variables, including the incidence of fetal distress, overall neonatal, maternal outcomes, and the efficacy of the admission test in predicting perinatal outcomes in both low- and high-risk pregnancies.

### Statistical Analysis

Statistical analysis of data was performed using Statistical Package for Social Sciences (SPSS) software version 21.0. Categorical variables were presented as numbers and percentages (%) and continuous variables as mean ± SD and median. Qualitative variables were correlated using the Chi-Square test. The diagnostic test was used to calculate sensitivity, specificity, negative predictive value (NPV), and positive predictive value (PPV). A p-value of < 0.05 was considered statistically significant.

### Ethical issues

The present research involving human subjects was conducted following the ethical standards of all applicable national and institutional committees and the World Medical Association’s Helsinki Declaration. It was conducted after informed written consent from the participants and ethical approval from the Institutional Ethical Committee (IEC number: 2015/571).

### Results

**Sociodemographic outcomes**: Of 100 term antenatal women admitted to the labour ward in the first stage of labour, 51 were low-risk and 49 were high-risk pregnancies. The common high-risk factors observed were hypertensive disorders of pregnancy (20.4%), anemia (20.4%), seizure disorders other than eclampsia (20.4%), gestational diabetes mellitus (16.3%), Rh-incompatibility (8.2%), oligohydramnios (6.1%), fetal growth restriction (4.1%) and post-date (4.1%). The mean age and gestation of women at the time of admission were 24.76 ± 2.98 years and 38.83 ± 1.09 weeks, respectively. The majority of the participants were multigravida (57%), and around 55% were booked. The various sociodemographic features of the participants are depicted in Table 1.

**Table 1:** Sociodemographic features

Parameters	Number (n)	Percentage (%)
**AGE (YEARS)**		
≥ 20–< 30	94	94.0%
≥ 30	6	6.0%
**REGISTRATION STATUS**		
Booked	55	55.0%
Unbooked	45	45.0%
**GESTATION (WEEKS)**		
≥ 37–< 40	98	98.0%
≥ 40	2	2.0%
**GRAVIDITY**		
Multigravida	57	57.0%
Primigravida	43	43.0%
**CATEGORISATION BASED ON RISK FACTORS**		
Low-risk group	51	51.0%
High-risk group	49	49.0%

**Obstetric outcome**: In the low-risk group, a total of 47 women had reactive, two (3.9%) suspicious and two (3.9%) ominous FHR tracing, whereas in the high-risk group, 20 (40.8%) participants had reactive, 16 (32.6%) suspicious, and 13 (26.5%) ominous FHR tracing on admission CTG (p < 0.0001), indicating the crucial role of admission CTG in the detection of fetal distress, especially in high-risk pregnancies. Of the total 100 participants, only five (5%) had meconium-stained fluid. The correlation between the colour of liquor and CTG results in both low- and high-risk pregnancies is depicted in Table 2. It was observed that the majority of participants with clear liquor had reactive CTG in both groups, whereas participants with meconium-stained fluid either had suspicious or ominous FHR tracing on CTG (p < 0.05). The majority of participants in both the groups with reactive CTG had a normal vaginal delivery (56%), and all the participants with ominous CTG tracing had an emergency cesarean section (100%). The association between the admission CTG results and the mode of delivery in the low-and high-risk group is depicted in Table 3.

**Table 2:** Relationship between the colour of liquor and CTG results in both low- and high-risk pregnancies.

	Admission CTG				
Meconium Staining	Reactive	Suspicious	Ominous	Total	p-value
**Low risk**					
Clear	47 (95.9%)	1 (2.04%)	1 (2.04%)	49	<0.001
Meconium-stained	0 (0%)	1 (50.0%)	1 (50.0%)	2	
Total	47 (92.2%)	2 (3.9%)	2 (3.9%)	51	
**High risk**					
Clear	20 (43.5%)	16 (34.8%)	10 (21.7%)	46	0.018
Meconium-stained	0 (0%)	0 (0%)	3 (100.0%)	3	
Total	20 (40.8%)	16 (32.7%)	13 (26.5%)	49	

**Table 3:** Relationship between the admission CTG results and the mode of delivery in the low-and high-risk groups

		Groups			
Admission CTG	Mode of Delivery	Low Risk	High Risk	Total	p-value
**Reactive**	ND	41 (87.2%)	15 (75.0%)	56 (83.6%)	
	LSCS	2 (4.3%)	1 (5.0%)	3 (4.5%)	
	Instrumental	4 (8.5%)	4 (20.0%)	8 (11.9%)	
	Total	47 (100.0%)	20 (100.0%)	67 (100.0%)	
**Suspicious**	ND	0(0%)	4 (25.0%)	4 (22.2%)	
	LSCS	1 (50.0%)	4 (25.0%)	5 (27.8%)	
	Instrumental	1 (50.0%)	8 (50.0%)	9 (50.0%)	
	Total	2 (100.0%)	16 (100.0%)	18 (100.0%)	0.0001
**Ominous**	ND	0(0%)	0 (0%)	0 (0%)	
	LSCS	2 (100.0%)	13 (100.0%)	15 (100.0%)	
	Instrumental	0(0%)	0 (0%)	0 (0%)	
	Total	2 (100.0%)	13 (100.0%)	15 (100.0%)	
**Total**	ND	41 (80.4%)	19 (38.8%)	60 (60.0%)	
	LSCS	5 (9.8%)	18 (36.7%)	23 (23.0%)	
	Instrumental	5 (9.8%)	12 (24.5%)	17 (17.0%)	
	Total	51(100.0%)	49 (100.0%)	100 (100.0%)	

**Perinatal outcome**: Of the total 100 neonates delivered to all the participants, the mean birth weight of all was 2.85 Kg. No significant correlation was observed between the admission test results and neonatal birth weight in both low- and high-risk groups. The correlation between one and five minutes of Apgar scores, neonatal resuscitation, NICU admission and admission CTG results is depicted in Table 4. A statistically significant correlation was observed between abnormal CTG results and adverse neonatal outcomes, including poor Apgar scores, need for neonatal resuscitation and NICU admission in both groups. The comparison of sensitivity, specificity, NPV and PPV of admission CTG in the prediction of neonatal outcomes between low- and high-risk pregnancies is depicted in Table 5. It was observed that the admission test has high specificity and NPV in the prediction of fetal distress in both low- and high-risk participants.

**Table 4:** Relationship between admission CTG and perinatal outcome

Groups	Parameters	Admission CTG			p-value
		Reactive	Suspicious	Ominous	
**Low-risk**			**Apgar score at 1 minute**		
	≥ 7	46 (97.9%)	1 (50.0%)	1 (50.0%)	0.0005
	< 7	1 (2.1%)	1 (50.0%)	1 (50.0%)	
			**Apgar score at 5 minutes**		
	≥ 7	47 (100.0%)	2 (100.0%)	2 (100.0%)	No correlation observed
	< 7	0 (0%)	0 (0%)	0 (0%)	
			**Neonatal resuscitation**		
	Yes	0 (0%)	0 (0%)	0 (0%)	No correlation observed
	No	47 (100.0%)	2 (100.0%)	2 (100.0%)	
			**NICU admission**		
	Yes	3 (6.4%)	1 (50.0%)	1 (50.0%)	0.019
	No	44 (93.6%)	1 (50.0%)	1 (50.0%)	
**High-risk**			**Apgar score at 1 minute**		
	≥ 7	17 (85.0%)	12 (75.0%)	5 (38.5%)	0.015
	< 7	3 (15.0%)	4 (25.0%)	8 (61.5%)	
			**Apgar score at 5 minutes**		
	≥ 7	20 (100.0%)	16 (100.0%)	13 (100.0%)	No correlation observed
	< 7	0 (0%)	0 (0%)	0 (0%)	
			**Neonatal resuscitation**		
	Yes	0 (0%)	1 (6.3%)	5 (38.5%)	0.03
	No	20 (100.0%)	15 (93.7%)	8 (61.5%)	
			**NICU admission**		
	Yes	3 (15.0%)	5 (31.3%)	10 (76.9%)	0.001
	No	17 (85.0%)	11 (68.7%)	3 (23.1%)	

**Table 5:** Comparison of sensitivity, specificity, negative and positive predictive value of admission CTG in the prediction of neonatal outcomes between low- and high-risk pregnancies.

Group	Parameter	Sensitivity (95% CI)	Specificity (95% CI)	Positive Predictive Value (95% CI)	Negative Predictive Value (95% CI)
**Low-risk**	Apgar score at 1-min	50.0% (1.26–98.7%)	97.9% (88.7–99.9%)	50.0% (1.3–98.7%)	97.9% (88.7–99.9%)
	NICU Admission	25.0% (0.6–80.6%)	97.8% (88.2–99.9%)	50.0% (1.3–98.7%)	93.6% (82.5–98.7%)
**High-risk**	Apgar score at 1-min	72.7% (39.0–93.9%)	77.3% (54.6–92.2%)	61.5% (31.6–86.1%)	85.0% (62.1–96.8%)
	NICU Admission	76.9% (46.2–94.9%)	85.0% (62.1–96.8%)	76.9% (46.2–94.9%)	85.0% (62.1–96.8%)

## Discussion

Admission CTG or the admission test is the most commonly used test for surveillance of fetal well-being, especially in high-risk pregnancies all over the world [8]. In the present prospective study, the FHR tracings on the admission test in low- and high-risk term antenatal women were observed in the relation to the overall perinatal and maternal outcomes. In our study, of the total 100 participants, 51 belonged to the low-risk group and 49 to the high-risk group with anemia (20.41%), hypertensive disorders of pregnancy (20.41%) and seizure disorders (20.41%) being the most common risk factors. A similar study conducted to assess the role of admission CTG as a predictor of the neonatal outcome included 67% multigravida and 34% primigravida women, and in their study also anemia (22%) was the most prevalent risk factor observed [5]. In the present study, the admission CTG was reactive in 67% (47 in low-risk and 20 in the high-risk group), suspicious in 18% (2 in low-risk and 16 in high-risk) and ominous in 15% (2 in low-risk and 13 in high-risk). In the low-risk group, 50% of participants with suspicious and ominous tracing on admission CTG had meconium-stained fluid, whereas in the high-risk group 100% of participants with ominous tracing had meconium-stained fluid. This was similar to the results of a study conducted on 160 term antenatal women, which reported that the admission CTG was found to be reactive in 77%, equivocal in 14.4% and ominous in 8.7% of women. They also reported that meconium-stained fluid was more common in women with ominous tracing (72%) [2]. Similar results were reported by other studies also, which found a significant correlation between non-reactive (suspicious/ ominous) admission CTG results and meconium-stained fluid [5, 9, 10, 11]. Another recent study conducted on 130 term high-risk antenatal women reported that the results of admission test were reactive in 73.1%, equivocal in 14.6% and ominous in 12.3%, and similar to our study they also observed a significant correlation between the results of admission CTG and mode of delivery with increased cesarean section rate in women with equivocal or ominous tracing [12]. Similar results of increased risk of operative delivery in mothers having pathological CTG were reported by a recent study [9].

In our study, a significant correlation was found between neonatal Apgar scores, need for resuscitation and NICU admission in neonates delivered to mothers with ominous or suspicious admission CTG results. Similar to our results, another study reported a significant correlation between abnormal (suspicious/ominous) admission test findings and poor Apgar scores at birth, need for neonatal resuscitation and increased risk of NICU admission (p = 0.000) [12]. A similar recent study found no significant correlation between admission CTG findings and the fifth-minute Apgar scores [13]. Other studies have also reported a significantly increased incidence of fetal distress, meconium-stained fluid, Apgar score < 7 and NICU admission in neonates delivered to mothers with pathological patterns on admission CTG tracing [11, 14, 15, 16].

In the present study, the admission CTG had high specificity and NPV in both low- and high-risk groups in the prediction of Apgar scores at birth (97.9% and 97.9% in the low-risk group vs. 77.3% and 85.0% in high-risk group) and NICU admission (97.8% and 93.6% in the low-risk group vs. 85.0% and 85.0% in high-risk group). A similar study reported a sensitivity of 44% specificity of 95% and a PPV of 50% for admission CTG in the prediction of 5-min Apgar scores [17]. Another systematic review reported that the admission CTG test had a specificity between 78% and 98%, and NPV between 67% and 99%, hence, indicating the role of admission CTG in the identification of women with no adverse outcomes [6].

## Conclusion

Admission CTG is a simple, easy, cost-effective, non-invasive screening method useful in the detection of fetal distress already present at the time of admission and can play a crucial role in predicting fetal well-being during labour. It also helps in planning early intervention so as to prevent adverse perinatal outcomes, especially in hospitals with limited resources and huge patient loads. The major drawback associated with admission CTG is that it increases the incidence of cesarean sections in places where fetal blood sampling is not performed to confirm fetal hypoxia during labour.

### Limitations

The present study was conducted for a short duration and with limited sample size. The number of high-risk cases included in the study was only 49. In the future, we can plan to conduct a multicentric study with a large sample size of only high-risk pregnant women. Furthermore, we will include fetal blood sampling to confirm fetal hypoxia before planning for emergency operative delivery, so as to reduce the risk of unnecessary cesarean sections.

**Key Points**

- The admission CTG was reactive in 67% (47 women in low-risk and 20 in the high-risk group), suspicious in 18% (2 in low-risk and 16 in high-risk) and ominous in 15% (2 in low-risk and 13 in high-risk) of participants.
- In the low-risk group, 50% of participants with suspicious and ominous tracing on admission CTG had meconiumstained fluid, whereas in the high-risk group 100% with ominous tracing had meconium-stained fluid.
- A significant correlation was observed between the results of admission CTG and mode of delivery with increased cesarean section rate in women with equivocal or ominous tracing.
- A statistically significant correlation was observed between abnormal CTG results and adverse neonatal outcomes, including poor Apgar scores, need for neonatal resuscitation and NICU admission in both low- and high-risk groups.
- No significant correlation was observed between the admission test results and neonatal birth weight.
- The admission CTG had high specificity and NPV in both low- and high-risk groups in the prediction of Apgar scores at birth (97.9% and 97.9% in the low-risk group vs. 77.3% and 85.0% in the high-risk group) and NICU admission (97.8% and 93.6% in the low-risk group vs. 85.0% and 85.0% in high-risk group).
